# Few Single Nucleotide Variations in Exomes of Human Cord Blood Induced Pluripotent Stem Cells

**DOI:** 10.1371/journal.pone.0059908

**Published:** 2013-04-01

**Authors:** Rui-Jun Su, Yadong Yang, Amanda Neises, Kimberly J. Payne, Jasmin Wang, Kasthuribai Viswanathan, Edward K. Wakeland, Xiangdong Fang, Xiao-Bing Zhang

**Affiliations:** 1 Department of Medicine, Loma Linda University, Loma Linda, California, United States of America; 2 CAS Key Laboratory of Genome Sciences, Beijing Institute of Genomics, Chinese Academy of Sciences, Beijing, China; 3 Division of Anatomy, Loma Linda University, Loma Linda, California, United States of America; 4 Center for Health Disparities and Molecular Medicine, Loma Linda University, Loma Linda, California, United States of America; 5 Department of Immunology and Genomics Core Facility, the University of Texas Southwestern Medical Center, Dallas, Texas, United States of America; French Blood Institute, France

## Abstract

The effect of the cellular reprogramming process *per se* on mutation load remains unclear. To address this issue, we performed whole exome sequencing analysis of induced pluripotent stem cells (iPSCs) reprogrammed from human cord blood (CB) CD34^+^ cells. Cells from a single donor and improved lentiviral vectors for high-efficiency (2–14%) reprogramming were used to examine the effects of three different combinations of reprogramming factors: OCT4 and SOX2 (OS), OS and ZSCAN4 (OSZ), OS and MYC and KLF4 (OSMK). Five clones from each group were subject to whole exome sequencing analysis. We identified 14, 11, and 9 single nucleotide variations (SNVs), in exomes, including untranslated regions (UTR), in the five clones of OSMK, OS, and OSZ iPSC lines. Only 8, 7, and 4 of these, respectively, were protein-coding mutations. An average of 1.3 coding mutations per CB iPSC line is remarkably lower than previous studies using fibroblasts and low-efficiency reprogramming approaches. These data demonstrate that point nucleotide mutations during cord blood reprogramming are negligible and that the inclusion of genome stabilizers like ZSCAN4 during reprogramming may further decrease reprogramming-associated mutations. Our findings provide evidence that CB is a superior source of cells for iPSC banking.

## Introduction

The discovery of a simple approach for reprogramming human somatic cells into induced pluripotent stem cells (iPSCs) has revolutionized regenerative medicine [1], [2], [3]. Technological breakthroughs have made it possible to generate integration-free iPSCs with modified mRNAs [4], [5], non-integrating Sendai virus [6], [7], [8], [9], [10] or oriP/EBNA1-based episomal vectors [11], [12], [13], [14], [15], [16], [17], [18], [19] and other methods, which brings iPSC-based therapy one step closer to clinical application. However, investigations into genetic aberrations, such as copy number variations (CNVs) and single nucleotide variations (SNVs) in iPSC genomes or exomes have identified exceedingly high-levels of genetic alterations in iPSCs generated from fibroblasts by various approaches [20], [21], [22], thus casting doubt on the future of iPSCs. In addition, iPSC lines have been found to harbor genetic alterations, particularly after long-term passage, similar to what has been observed for embryonic stem cells (ESCs) [23], [24], [25], [26]. In contrast to earlier publications, a more recent study suggests that cellular reprogramming may not be mutagenic *per se* and that the observed SNVs are merely the fixation of pre-existing rare mutations in the parental cell pool [27]. These seemingly conflicting reports warrant further investigation into whether the process of iPSC generation is mutagenic and if so, the extent of such mutations.

Three mechanisms have been proposed to account for the up to 10-fold higher rate of genetic alterations in iPSCs as compared to anticipated background mutations. First, the fixation of rare mutations in the parent cell population has been implicated. Early studies suggest that ∼50% of SNVs are pre-existing in parent cell cultures [20]. A recent report demonstrates that 30% of skin fibroblasts have somatic CNVs in their genomes [28]. Second, the selection of clones harboring mutations that improve reprogramming efficiency and/or promote cell survival/proliferation has been suggested as a contributing factor. This idea is supported by enrichment analysis that found that the observed genetic variations are strongly associated with cancer [20]. A third proposed mechanism is proliferative stress induced by reprogramming factor overexpression. In support of this hypothesis, some reprogramming factors such as MYC are strongly oncogenic [29]. Furthermore, the downregulation of genome guardians like p53 substantially increases reprogramming efficiency [15], [19], [30], [31].

A careful examination of reported data suggests that several factors might affect the number of SNVs identified in the coding regions of each iPSC clone. First, reprogramming efficiency is a potential factor. Extremely low reprogramming efficiency (10^−6^) is associated with very high levels of SNVs (more than 10 per iPSC) [32], [33]. Thus, low reprogramming efficiency might also contribute to the outgrowth of clones with mutations in genes that promote cell growth and exert causative effects in cancer [20]. Second, long-term culture may lead to the accumulation of rare SNVs, since longer durations of in vitro culture after harvest of the primary cells is associated with increased numbers of SNVs [27], [32]. Third, source cells from reprogramming may also play a role: hematopoietic CD34^+^ cell-derived iPSCs harbor less than half the mutations detected in iPSC clones from MSC or fibroblasts [32].

Given the potential contribution of the above factors, we propose that an accurate estimate of reprogramming-induced SNVs requires the use of a high-efficiency approach (>1%) for the reprogramming of homogenous primary cells from a single donor with minimal in vitro manipulation. The majority of CD34^+^ hematopoietic stem/progenitor cells in adults reside in the bone marrow niche and are protected from environmental insults, thus are presumably more homogeneous than fibroblasts from skin biopsy [28], [32]. Umbilical cord blood (CB) is a source of CD34^+^ hematopoietic cells that is superior to and more homogeneous than adult blood or marrow cells. This is because CB is a source from earlier in life and the pool of CD34^+^ cells in the baby has been less extensively expanded than adult blood or marrow [34], [35]. Thus, CB CD34^+^ cells are less likely to harbor unique rare mutations than cells from other sources. In addition, we recently reported that CB CD34^+^ cells can be very efficiently reprogrammed to iPSCs (2%) using improved lentiviral vectors [18], thus providing us with the unique opportunity to address an important and largely unanswered question: What is the contribution of reprogramming *per se* to genetic alterations in iPSC?

## Materials and Methods

### Cord Blood

The use of CB was approved by the Institutional Review Board of Loma Linda University and written informed consent was obtained from all participants. After treating CB with red blood lysis buffer, CD34^+^ cells were purified from nucleated cells by MACS (Miltenyi Biotec, Auburn, CA). All the iPSC clones for exome sequencing analysis were derived from a single CB.

### Constructs and Lentiviral Vector Packaging

In conducting work involving the use of recombinant DNA, we adhered to the current version of the National Institutes of Health (NIH) Guidelines for Research Involving Recombinant DNA Molecules. The lentiviral vector constructs have been detailed previously [36]. In brief, a strong promoter SFFV was used to drive the expression of OS (*OCT4* and *SOX2*) or MK (*MYC* and *KLF4*), which are linked with a 2A self-cleavage peptide sequence [18], [37]. Vector containing the *ZSCAN4* gene was obtained from Applied Biological Materials Inc. (ABM; Richmond, BC, Canada). Detailed methods for lentiviral vector packaging and titering have been published [36]. After a 100-fold concentration by ultracentrifugation, biological titers of 5–10×10^7^ were achieved.

### iPSC Generation

CB CD34^+^ cells were cultured in hematopoietic cell culture conditions: Iscove’s modified Dulbecco’s medium (IMDM)/10% FBS supplemented with cytokines TPO, SCF, FL and G-CSF each at 100 ng/ml, and IL-3 at 10 ng/ml [38], [39]. After 2 days of pre-stimulation, 1×10^4^ cells per well were seeded into a CH-296 (Takara Bio, Inc., Shiga, Japan)-treated non-TC 24-well plate. Lentiviral vectors were added at an MOI of 4 and co-cultured for 16 hours. Protamine sulphate at a final concentration of 8 µg/ml was added to increase the transduction efficiency. After transduction, cells were harvested and transferred to 6-well plates pre-seeded with inactivated rat embryonic fibroblast (REF) feeder cells (ABM). Cells were maintained in the hematopoietic cell culture condition for 2 more days before being gradually replaced with iPSC media. The iPSC medium used in our study is composed of Knockout DMEM/F12 medium (Invitrogen; Carlsbad, CA) supplemented with 20% Knockout Serum Replacement (KSR) (Invitrogen), 1 mM GlutaMAX (Invitrogen), 2 mM nonessential amino acids (ABM), 1×penicillin/streptomycin (ABM), 0.1 mM β-mercaptoethanol (Sigma-Aldrich Corp, St. Louis, MO), 20 ng/ml FGF2 (ABM), and 50 µg/ml ascorbic acid [40], [41]. To increase reprogramming efficiency, sodium butyrate [42], [43] was added at 0.25 mM from day 2 to 10, and cells were cultured under hypoxia [44], [45].

### Flow Cytometry

iPSCs were harvested with Accutase (Innovative Cell Technologies, Inc., San Diego, CA) and fixed for 10 min at room temperature in fixation buffer (eBioscience, Inc., San Diego, CA). For staining with TRA-1-60-PE (eBioscience), cells were incubated with the antibody for 30 min at room temperature. Flow cytometry analysis was performed using a FACS Aria II (BD Biosciences, San Jose, CA) with a 488-nm laser. 30, 000 events were collected for each sample. For flow cytometry analysis, gates were set based on isotype controls.

### Confocal Imaging

For immunostaining of iPSC colonies, iPSCs were cultured in 2-well chamber culture slides for 4–5 days. Cells were treated with fixation buffer supplemented with permeabilization buffer (eBioscience) for 10 min before being stained overnight with PE or FITC conjugated antibodies OCT4 (eBioscience), NANOG (BD), or SSEA4 (eBioscience). The samples were washed twice with permeabilization buffer, and then coverslipped. Imaging was performed using the Zeiss LSM 710 NLO laser scanning confocal microscope with a 20× objective at the Loma Linda University Advanced Imaging and Microscopy Core. High resolution monochrome image was captured using a Zeiss HRm CCD camera.

### Teratoma Assay

The use of NOD/SCID/IL2RG^−/−^ (NSG) immunodeficient mice for the teratoma formation assay was approved by the Institutional Animal Care and Use Committee at Loma Linda University (LLU). NSG mice were purchased from the Jackson Laboratory and maintained at the LLU animal facility. iPSCs were harvested by Dispase (Invitrogen) digestion, and approximately 1×10^6^ iPSCs were re-suspended in 200 µl DMEM/F12 diluted (1∶1) Matrigel solution (BD) before subcutaneous injection into NSG mice. At 2 months after injection of iPSCs, teratomas were dissected and fixed in 10% formalin. After paraffin embedding and microsectioning, samples were stained with hematoxylin and eosin (H & E), following standard protocol. Pictures of differentiated tissues were captured with a Nikon microscope using a 20× objective.

### Exome Sequencing

To deplete feeder cells, iPSCs were cultured in TeSR medium (StemCell Technologies) for 1 passage before cell harvest. Genomic DNA from passage 5 iPSCs was extracted using the Gentra Puregene Cell Kit (Qiagen). Libraries were prepared using the Illumina TruSeq DNA Sample Prep Kit. In brief, DNA was fragmented (∼200–350 bp) and ligated to the Illumina sequencing adaptor oligonucleotides. The adaptor-ligated fragments were amplified by PCR and then hybridized to the Illumina TruSeq Exome Enrichment Kit, which covers 1.22% of human genomic regions corresponding to the CDS (coding sequence) exons. The hybridized fragments were captured by streptavidin-coated magnetic beads, followed by sequencing on a Hiseq2000 sequencer using 100-bp paired-end reads. The image analysis and base calling were performed using the Illumina pipeline (v1.8) with default settings.

### Bioinformatic Analysis

All reads were aligned to human reference sequence (release hg19, Feb. 2009) from University of California - Santa Cruz (UCSC) with the Burrows-Wheeler Aligner (BWA) version 0.6.1-r104 [46]. Picard version 1.57 was used to convert, sort, and index the aligned data files and remove PCR duplicates. For discovery of variations, we implemented a pipeline based on the Genome Analysis Toolkit (GATK) version 1.6–9 [47]. First, sequence reads were locally realigned and base-quality scores recalibrated. Second, variants were identified by the Unified Genotyper program in GATK. Third, low-quality variants were filtered using the Variant Filtration Walker tools in GATK and in-house developed codes. A minimum read depth of five and consensus quality of 50 was required at every examined location. Variants flanking homopolymer longer than 5 were removed. Any three or more variants located in a 50-bp window were discarded. Variants that had a record in the dbSNP database (version 135) were removed from consideration to reduce the false-positive rate [20]. For the heterozygous sites, both normal and variant depth should be more than five. For the homozygous sites, normal depth should be less than 1 and variant depth should be greater than 5. Variants that occurred in all the iPSC lines were removed from consideration. The filtered variants were annotated with ANNOVAR [48] and the effect of each variant was predicted with SIFT [49].

### Verification of SNVs

To validate SNVs identified by bioinformatic analysis, we used a real-time PCR approach. We designed 3 primers for each point mutation. Two forward primers, with SNV site at the 3′ end, were manually designed and had melting temperatures of 50–55°C. One forward primer matches the wildtype allele, while another matches the SNV allele. The reverse primer was designed using Primer3Plus (http://frodo.wi.mit.edu/) with a melting temperature of 60°C. Equal amount of DNA (100 ng) was used for the sample that harbors a particular SNV and 4 controls that do not. Real-time PCR was performed using SYBR® Green PCR Master Mix (Applied Biosystems, Foster City, CA) on the 7500 Fast Real-Time PCR System (Applied Biosystems). The amplification program consisted of 50°C for 2 min and 95°C for 10 min, and was followed by 40 cycles of 95°C for 15 sec and 60°C for 1 min. ΔCt was calculated by subtracting Ct cycles when SNV and wildtype primers were used. Because the SNV primer can amplify the SNV allele more efficiently, leading to lower Ct cycle number, comparison of the two Cts can identify samples with or without the particular SNV. To prevent false positives, we arbitrarily call positive for the SNV with ΔCt of more than 1.

### Ingenuity Pathways Analysis

To examine significantly over-represented networks and pathways, we analyze all the identified SNVs pooled from all the iPSC clones by Ingenuity Pathways Analysis (IPA, Ingenuity® Systems, www.ingenuity.com). Ingenuity knowledge base is the largest manually curated database for pathway analysis [50].

### COSMIC Analysis

To test if the genes harboring variants occurred during the reprogramming process are enriched in gene set bearing cancer-associated mutations, we queried Catalogue of Somatic Mutations in Cancer (COSMIC) v62 (http://www.sanger.ac.uk/genetics/CGP/cosmic/).

### Statistics

Data are presented as mean ± standard deviation (SD). Two-tailed Student t test was performed. *P* values of <0.05 were considered statistically significant.

## Results

### Generation of iPSCs from CB CD34^+^ Cells with Three Different Combinations of Reprogramming Factors

We are interested in reprogramming CB CD34^+^ cells, because CB has been proposed as a cell source in iPSC banking for allogeneic cell replacement therapy and CB may possess fewer genetic mutations than skin fibroblasts and PB [35]. To minimize the likelihood of clonal selection in low-efficiency reprogramming, we used lentiviral vectors to reprogram CB. Using an lentiviral vector optimized to achieve high-level transgene expression in hematopoietic cells [18], we have been able to reprogram 2% CB CD34^+^ cells into iPSCs with OCT4 and SOX2 (OS) alone, an efficiency that is ∼1000-fold higher than previously reported [34]. This ability allowed us to compare the effects of different combinations of reprogramming factors on SNV loads in iPSC clones without the confounding effect of low reprogramming efficiency. For this purpose, we generated iPSCs using OS alone, using OS and ZSCAN4 (abbreviated as OSZ or Z for simplicity), or using OS and MK (abbreviated as OSMK or MK for simplicity). ZSCAN4 was used in combination with OS because it has been shown to enhance telomere lengthening, regulate genomic stability, and improve the quality of iPSCs [51], [52], [53]. The Yamanaka combination, OSMK, served as a control in our experiments since it has been employed in the majority of previous iPSC exome sequencing studies [20], [22], [54].

**Figure 1:**
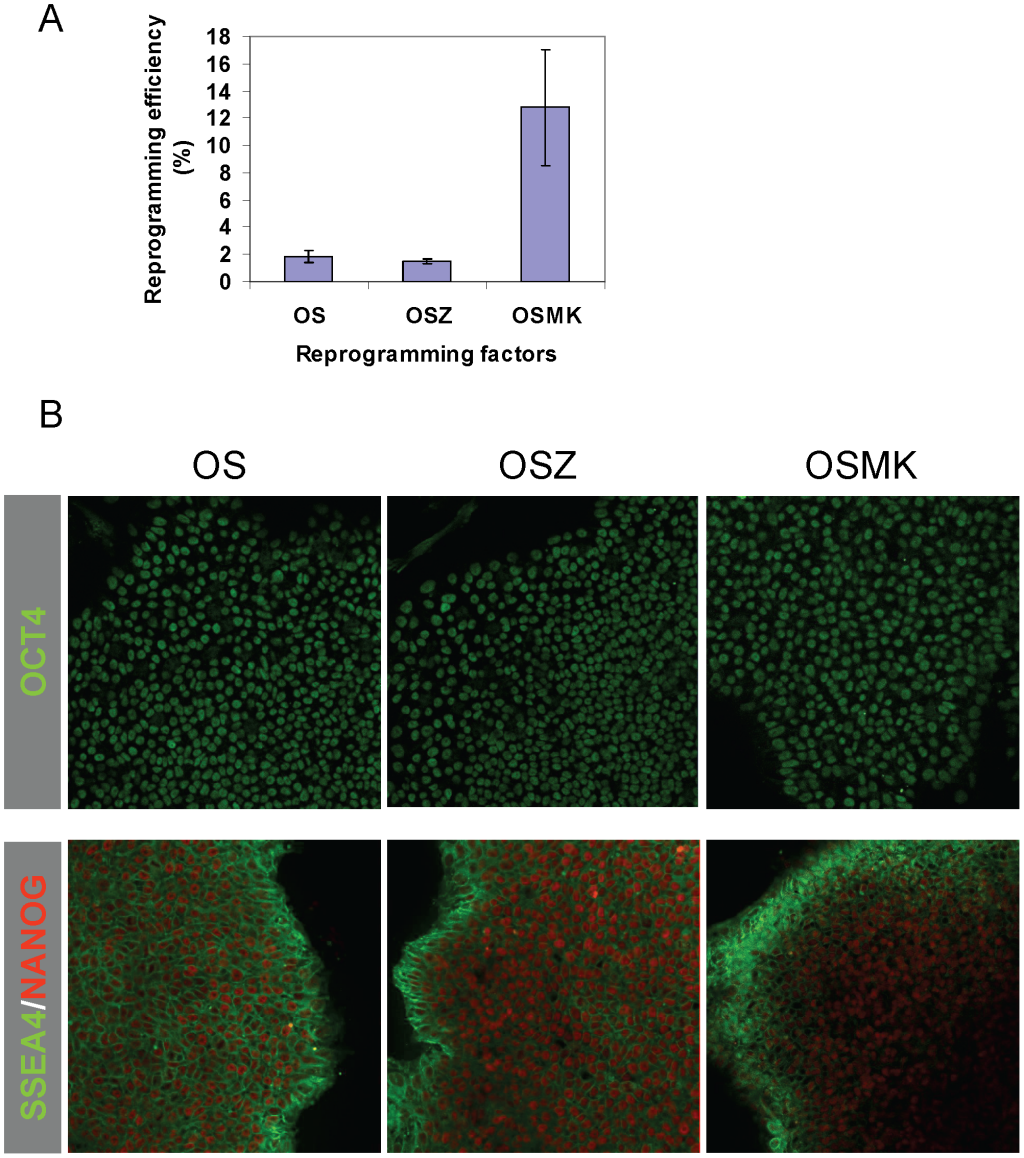
Efficient generation of iPSCs from cord blood CD34^+^ cells. (**A**) Efficient reprogramming of cord blood with lentiviral vectors. Three different combinations of reprogramming factors were used: OCT4 and SOX2 (OS), OS+ZSCAN4 (OSZ), and OS+MYC and KLF4 (OSMK). After transduction, 2500–5000 cells were seeded in 6-well plates. iPSC colonies were counted 2 weeks later and reprogramming efficiencies were calculated accordingly. (**B**) iPSCs express pluripotency makers OCT4, NANOG and SSEA4. Representative pictures for each group (OS iPSC lines, OSZ iPSC lines, and MK iPSC lines (OSMK)) are presented (200×). Confocal imaging did not identify any differences in expression of pluripotency makers among 15 iPSC lines.

To minimize the accumulation of random mutations during long-term in vitro culture, we cultured CB CD34^+^ cells for only 2 days before lentiviral transduction. Consistent with our previous report, we found that 2% of CB CD34^+^ cells can be reprogrammed into iPSCs with OS (**Figure 1A**). However, in contrast to early studies [52], [53], inclusion of ZSCAN4 appeared to decrease the reprogramming efficiency, albeit not reaching statistical significance (n = 3, *P* = 0.2; **Figure 1A**). This result is reminiscent of our early finding that KLF4, alone, does not increase OS-mediated reprogramming, likely because OS-mediated reprogramming is highly efficient [18]. However, the inclusion of both MYC and KLF4 (MK), expressed in a single vector, substantially increased reprogramming efficiency to 14% (n = 3, *P*<0.05 compared to OS or OSZ; **Figure 1A**).

To accurately compare SNVs in each iPSC clone, we picked iPSC colonies generated from a single cord blood. Most iPSC clones were able to be passaged long-term and maintained typical iPSC morphology. We randomly selected 5 clones from each group for further analysis. No obvious differences were observed in the expression of pluripotency markers like OCT4, NANOG, SSEA4 and TRA-1-60 after passage 10 (**Figure 1B** and **Figure S1**). However, we did observe that higher portions of cells express TRA-1-60 at passages 3 in OSZ compared to OS iPSC clones (62±9% vs. 42±8%; n = 5; *P*<0.01), suggesting that ZSCAN4 can increase the quality of OS-mediated reprogramming. This result is consistent with reports showing that inclusion of ZSCAN4 improves the quality of mouse iPSCs [52], [53]. To further characterize the iPSC clones, we performed teratoma assays. Histological analysis showed that teratomas generated from all of the 15 iPSC lines consisted of tissues from three germ layers such as cartilage, gut-like structures, neurotubules, and pigmented epithelial cells (**Figure S2ABC**). Taken together, these data demonstrate that the 15 iPSC clones are *bona fide* pluripotent stem cells.

### Exome Sequencing

To evaluate SNVs, we focused our analysis on mutations accumulated during reprogramming only, thus iPSCs at passage 5 were used. To minimize contamination of feeder cells, iPSCs were cultured in TeSR medium for 1 passage before harvest. To prevent unintended bias during cell culture, sample processing, exome capture and sequencing, all the 15 iPSC clones were cultured and processed in tandem. We enriched for protein coding genes using Illumina TruSeq Exome Enrichment Kit and sequenced the captured DNA from 15 samples using Illumia Genome Analyzer IIx with one sample per lane. After aligning the reads to the reference human genome (release hg19), we obtained 37–80 million uniquely aligned reads per sample (Table 1).

**Table 1:** Summary of the exome sequencing data and the identified single nucleotide variants.

iPSC lines	# Total reads (M)	# Unique reads (M)	# Heterozygous variants	% in dbSNP	Reads of variants
MK4	130.9	54.5	6150	97	23
MK5	127.1	51.8	24943	97	50
MK7	125.0	51.3	24907	97	50
MK8	143.4	58.7	25935	97	54
MK9	194.6	79.8	28150	97	72
OS1	89.6	36.8	19400	97	38
OS3	142.5	58.4	26142	97	56
OS5	141.0	58.0	25753	97	54
OS6	143.8	59.2	26247	97	55
OS7	124.8	51.2	24425	97	49
Z1	148.7	61.2	26187	97	59
Z2	126.7	52.2	23747	97	52
Z5	153.8	63.1	26277	97	61
Z6	154.3	62.8	26422	97	62
Z7	145.3	60.1	26046	97	59

The numbers of heterozygous variants are those that have a minimum of 5× coverage. The dbSNP percentage represents the portion of identified variants present in the Single Nucleotide Polymorphism Database.

We searched for single base changes, small insertions/deletions and alternative splicing variants and identified more than 20,000 known and novel variants that had a minimum read depth of five and consensus quality of 50 for the majority of iPSC lines (Table 1). An iPSC variant is defined as a mutation if it is present only in one clone and absent in other iPSC lines. We reason that if there is a rare preexisting SNV fixed in 1 out of 15 iPSC clones, this SNV is unlikely to be detectable in the parent CB sample, because we set the algorithms to call an SNV positive only if it is present in more than 10% reads. Given this, we did not sequence the parental sample. We identified 548 heterozygous novel SNVs shared by all of the samples, indicating that they were pre-existing variants in the parent CB sample. In contrast to earlier report that some samples share the same SNVs, we found that none of SNVs in our study was shared by 2 or more out of 15 iPSC clones, suggesting that CB CD34^+^ cells are very homogenous and that our identified SNVs are unlikely to arise from rare pre-existing variants. We also identified 34 SNVs that were unique to specific clones.

### Verification of SNVs

To verify the 34 SNVs identified by bioinformatics (Table 2), we developed a real-time PCR approach, which compared the differences in amplification efficiency when using a matched and a one-nucleotide mismatched primer at the 3′ end. This approach is demonstrated in **Figure S3**. The presence of a particular SNV led to more efficient PCR amplification when the relevant primer was used. When the difference amplification cycle or ΔΔCt was more than 1, the SNV was validated. We analyzed all the identified SNVs and 74% were verified by real-time PCR (Table S1). Due to technical limitations, SNVs that are present in 10% or less cells or located in repeat regions of the genome may not be validated. Some of the unvalidated SNVs may be false positives. However, to prevent underestimation of SNVs in CB iPSC lines, we pooled all the SNVs identified in any of the 5 iPSC clones generated with a particular factor combination for the following analyses.

**Table 2:** Genes found to be mutated in exomes of 15 CB iPSC lines.

iPSC line	Chrom	Position	Ref	Alt	Codon	Substitution	Region	SNP Type	Gene Name	COSMIC gene
MK4	chr2	160086962	G	A	ATG-ATa	M1569I	CDS	NS	TANC1	No
MK4	chr1	237713868	C	A	CGC-aGC	R1029S	CDS	NS	RYR2	No
MK4	chr3	118620274	C	A	–		3′UTR		IGSF11	No
MK5	chr6	4130793	T	C	GAA-GgA	E75G	CDS	NS	ECI2	No
MK5	chr20	61476966	G	T	–		3′UTR		DPH3P1	No
MK5	chr17	39305800	T	A	AGC-tGC	S74C	CDS	NS	KRTAP4-5	No
MK7	chr1	205819074	A	T	TTC-aTC	F43I	CDS	NS	PM20D1	No
MK7	chr12	48866491	C	T	ACG-AtG	T15M	CDS	NS	ANP32D	No
MK8	chr6	159054862	A	T	–		3′UTR		TMEM181	No
MK8	chr10	96022426	C	T	AAC-AAt	N1330N	CDS	S	PLCE1	No
MK9	chr9	23691717	G	T	–		3′UTR		ELAVL2	No
MK9	chr19	4910814	C	T	–		5′UTR		UHRF1	No
MK9	chr6	105548487	A	G	–		Downstream		BVES	No
MK9	chr15	43890440	G	A	CGT-CaT	R309H	CDS	NS	CKMT1B	No
OS1	chr12	53879244	G	A	TAC-TAt	Y279Y	CDS	S	MAP3K12	No
OS1	chr2	202010131	C	T	CTT-tTT	L117F	CDS	NS	CFLAR	No
OS1	chr19	12662311	G	C	–		5′UTR		ZNF564	No
OS3	chr5	156899405	C	T	CGT-tGT	R280C	CDS	NS	NIPAL4	No
OS3	chr14	24424340	G	A	ACG-ACa	T57T	CDS	S	DHRS4	No
OS3	chr17	39346592	ACCT	A	455_457delCCT	T152_C153>S	CDS	NS	KRTAP9-1	Yes
OS3	chr19	5838781	C	T	–		5′UTR		FUT6	No
OS3	chr19	58944373	C	G	–		3′UTR		ZNF132	No
OS5	chr4	154216795	G	A	GTC-aTC	V373I	CDS	NS	TRIM2	No
OS5	chr12	123875282	G	A	GAA-aAA	E80K	CDS	NS	SETD8	No
OS6	chr3	101546808	A	G	–		3′UTR		NXPE3	No
Z1	chr6	51482793	G	T	–		3′UTR		PKHD1	No
Z1	chr7	92238910	G	A	–		3′UTR		CDK6	No
Z2	chr4	140811131	G	T	CAA-aAA	Q26K	CDS	NS	MAML3	No
Z5	chr19	7992965	G	T	GTC-GTa	V375V	CDS	S	TIMM44	No
Z5	chr19	30503231	C	A	CGC-CGa	R366R	CDS	S	C19orf2	No
Z6	chr1	6314975	C	T	–		5′UTR		GPR153	No
Z6	chr12	70047490	G	A	–		3′UTR		BEST3	No
Z6	chr12	131438772	G	T	–		5′UTR		GPR133	No
Z7	chr16	683298	G	A	CCG-CCa	P296P	CDS	S	WFIKKN1	No

The full details of each SNV including reads of SNV and wildtype alleles are in Table S1.

MK: iPSC lines generated with OCT4, SOX2, MYC and KLF4; OS: iPSC lines generated with OCT4 and SOX2; Z: iPSC lines generated with OCT4, SOX2 and ZSCAN4.

CDS: coding sequence; UTR: untranslated region; Downstream: SNV is at downstream of 5′UTR; S: synonymous coding mutation; NS: nonsynonymous coding mutation.

### Few SNVs in Exomes of CB iPSCs and OSZ Appears to be a Better Combination for Generating iPSCs Harboring Fewer SNVs

**Figure 2:**
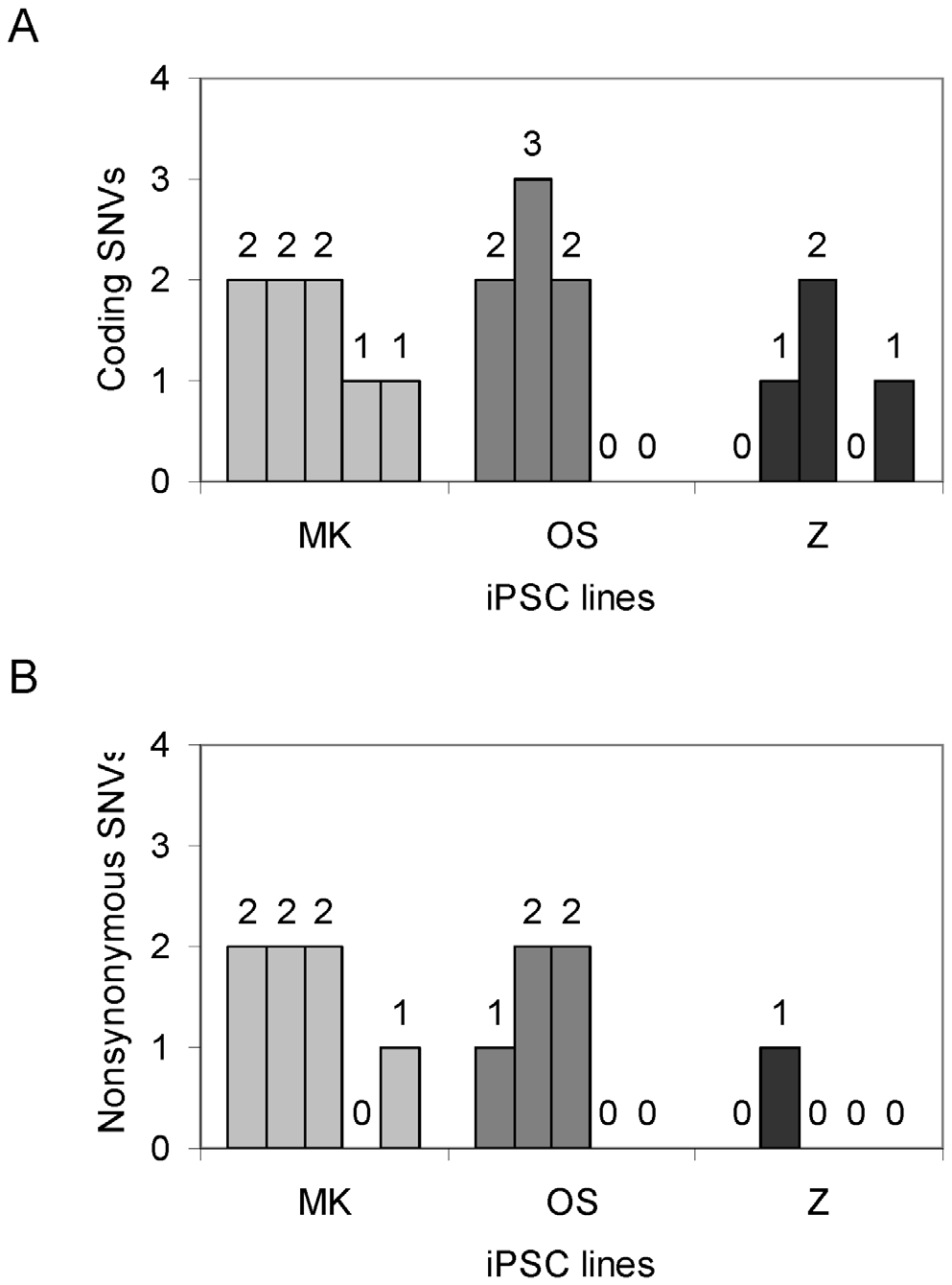
Coding mutations in each of the 15 CB iPSC lines. Number of coding SNVs (**A**) and nonsynonymous coding SNVs (**B**) in each line compared to the parent cord blood cells. MK: iPSC lines generated with OCT4, SOX2, MYC and KLF; OS: iPSC lines generated with OCT4 and SOX2; Z: iPSC lines generated with OCT4, SOX2; and ZSCAN4. The use of OSZ appears to decrease the coding SNV load in iPSC lines compared to the OSMK control (*P*>0.05) (**A**), while OSZ iPSC lines harbor significant fewer number of nonsynonymous coding SNVs relative to the OSMK control (*P*<0.05) (**B**).

As shown in Table 2, we identified 14, 11, and 9 SNVs on exomes including untranslated regions (UTR) in five clones of OSMK, OS, and OSZ iPSC lines. Among them, there are only 8, 7, and 4 protein-coding mutations in the 3 groups of iPSC lines. There is a trend that OS iPSC lines appear to harbor fewer SNVs than OSMK iPSC lines, and inclusion of Z to OS further decreases SNV loads during reprogramming, but the differences are not statistically significant. In each clone, 1.3 (range: 0–3) coding mutations was identified, which is remarkably lower than 5–10 SNVs identified in previous studies using fibroblasts and low-efficiency reprogramming approaches. Of note, 2 out of 5 OS or OSZ iPSC lines did not acquire any coding mutations during reprogramming (**Figure 2A**).

Synonymous SNVs do not alter amino acid sequence and thus may not be harmful to the cells. Accordingly, we also analyzed nonsynonymous SNVs. Of significant interest, only 1 nonsynonymous SNV was observed in OSZ iPSC lines compared to 7 and 5 for MK and OS iPSC clones (**Figure 2B**). OSZ iPSCs harbor significantly fewer nonsynonymous SNVs than OSMK iPSCs (0.2 vs. 1.4; *P*<0.05). This result suggests that the combination of OSZ may be used to generate “safer” iPSCs with fewer potentially risky SNVs than the commonly used OSMK factors.

### Pathway Analysis

Due to limited numbers of SNVs in each group, we combined all the 34 SNVs from 15 iPSC lines for analysis. Ingenuity Pathway analysis showed that the top network is cell development, cell growth and proliferation, hair and skin development and function (**Figure S4**). This result suggests that some SNVs might have improved iPSC proliferation.

To determine whether the genes identified with reprogramming-associated mutations are associated with cancer, we interrogated COSMIC, a database of genes commonly mutated in cancer. Only one out of 34 SNVs was found in this database, which is remarkably lower than the 50 out of 124 SNVs identified in the early report [20].

## Discussion

Here we report that CB iPSCs harbor an average of 1.3 coding mutations per line. The SNV load appears to be dependent on factors used during reprogramming: Each OSMK iPSC lines showed 1.6 protein-coding mutations, while OSZ iPSCs only acquired 0.8 such variations per line. In comparison, previous studies reported an average of 5–10 coding SNVs per iPSC line, a mutation rate that is estimated to be ∼10-fold higher than background mutation during in vitro culture [20], [22], [27], [32], [33]. For the first time, we observed SNVs acquired during iPSC generation that is similar to or only slightly higher than that expected by random mutation. In addition, our novel finding that genome stabilizers like ZSCAN4 can significantly decrease genetic mutation rates during reprogramming should have important implications for the clinical application of cellular reprogramming.

Several factors might have contributed to exceeding low SNV loads that we report in our 15 CB iPSC lines. First, SNVs in previous studies may have been overestimated. Several studies have concluded that 50% or even the majority of identified SNVs are pre-existing in fibroblasts. However, the number of pre-existing SNVs may still be an underestimate because the rare mutations (such as those occurred at the rate of 10^−6^) in skin fibroblasts and acquired during in vitro culture are unlikely to be detectable by current technologies [20], [27], [28]. Second, it is possible that SNVs in our study were underestimated. This is unlikely, because we intentionally decreased the required reads for SNVs from 10 in many studies to 5 to prevent false negative results. Accordingly, we identified >20,000 unique SNVs in the majority of our 15 iPSCs clones, which are substantially higher than many other studies [20], [22], [54]. A third possibility is that SNVs were reduced by our high-efficiency reprogramming approach. We converted 2–14% of transduced CB CD34^+^ cells into iPSCs, an efficiency that is 100–10,000 fold higher than those reported in similar studies. This high reprogramming efficiency would reduce the possibility that iPSCs were generated from cells selected because they harbored SNVs favorable for reprogramming. In addition, the duration from gene transduction to reprogramming initiation or the first cell division was 3–7 days in our study, compared to ∼2 weeks for fibroblast reprogramming. This may also have decreased chances for CB cells to accumulate more SNVs during seemingly quiescent stage after transduction of reprogramming factors.

We designed our experiments with aims to address two questions: 1) whether omitting MYC and KLF4 in the reprogramming combination can decrease SNV loads, and 2) whether genome stabilizer ZSCAN4 can decrease SNV loads. We did observe lower numbers of total SNVs and coding SNVs in OS iPSCs relative to OSMK iPSCs. But the differences are far from significant, suggesting that transient expression of MYC and other factors during reprogramming does not significantly increase mutation rates. With regard to ZSCAN4, we did observe a trend toward decreased coding SNVs from 1.6 in OSMK iPSCs to 0.8 in OSZ iPSCs. However, this difference did not reach statistical significance (*P* = 0.11), largely because the sample size was still small. However, nonsynonymous SNVs are significantly lower in OSZ iPSC lines compared to OSMK iPSC lines, suggesting that ZSCAN4 does play a positive role in stabilizing the genome and decreasing mutations during reprogramming. Taken together, these data suggest that the optimization of iPSC derivation conditions, through combinations of reprogramming factors and culture conditions, promotes genetic stability of pluripotent stem cells.

Because we identified few coding mutations in each clone of iPSCs, it was not our intent to determine how many of these SNVs were pre-existing in the cord blood sample. We cannot completely exclude the possibility that one or more of the identified SNVs are preexisting rare mutations in the parent CB CD34^+^ cell population. However, given that our reprogramming efficiency is 100–10,000 fold higher than that in the earlier studies and that CB CD34^+^ cells are much more homogenous than skin fibroblasts, we believe that almost all the identified SNVs are *de novo* mutations that occurred during reprogramming.

Our data suggest that reprogramming of CB CD34^+^ cells into iPSCs is not mutagenic, particularly when a genome stabilizer is included during reprogramming. However, this conclusion does not necessarily suggest that reprogramming of other types of cells like fibroblasts is not mutagenic. Cells that are difficult to reprogram such as fibroblasts are likely to result in increased mutations as compared to reprogramming of CB CD34^+^ cells, because clones that harbor mutations favorable for reprogramming are selected for and extended period of culture required for reprogramming increases chances for the accumulation of random mutations. Our data, together with these considerations, suggest that cord blood would be the best choice of cells for iPSC banking [35], [55], [56].

Taken together, our data demonstrate that it is possible to achieve reprogramming to full pluripotency with a very low level of SNV load that is close to the rate of random background mutation. Our finding that the genome stabilizer ZSCAN4 decreases coding mutation rates deserves further investigation on a large scale through whole genome sequencing.

## Supporting Information

Figure S1 **Flow cytometry analysis of CB iPSC lines.** FACS diagrams show the expression of the pluripotency factor TRA-1-60 on bulk populations of 15 CB iPSC lines cultured with feeder support. OS, iPSCs generated with OS alone; Z, iPSCs generated with OSZ; MK, iPSCs generated with OSMK.
(TIFF)

Figure S2 **Teratoma formation from CB iPS cells.** iPS cells were subcutaneously injected into NSG mice. After ∼2 months, the teratomas were analyzed by haematoxylin and eosin staining. (**A**) iPSCs generated with OS formed teratomas consisted of cartilage (mesoderm), gut-like structures (endoderm), and neurotubules (ectoderm). (**B**) iPSCs generated with OSZ (Z) formed teratomas consisted of cartilage (mesoderm), gut-like structures (endoderm), and neurotubules and pigmented epithelium (ectoderm). (**C**) iPSCs generated with OSMK (MK) lines formed teratomas consisted of cartilage (mesoderm), gut-like structures (endoderm), and neurotubules and pigmented epithelium (ectoderm).
(TIFF)

Figure S3 **Validation of SNV by real-time PCR.** (**A**). No obvious difference was observed when a pair of wildtype primers was use to amplify DNA from 5 iPSC lines. (**B**) When the primer harboring the SNV at the 3′ end was used, the sample DNA containing the particular SNV amplified more efficiently, leading to lower cycles. (**C**) In samples that ΔCt is substantially lower than the control, the SNV is validated (Left). However, in samples that ΔCt is not significantly lower than control (ΔΔCt <1), the SNV is not validated (Right).
(TIFF)

Figure S4 **Ingenuity pathway analysis of all the 34 SNVs identified in 15 iPSC lines.**
(TIFF)

Table S1 **Detailed information of genes found to be mutated in exomes of 15 CB iPSC lines.**
(XLS)
