# Supplementary figures and images for: Few Single Nucleotide Variations in Exomes of Human Cord Blood Induced Pluripotent Stem Cells
Source: PLoS One. 2013 Apr 1;8(4):e59908. doi: 10.1371/journal.pone.0059908 (PMC3613421; doi:10.1371/journal.pone.0059908)

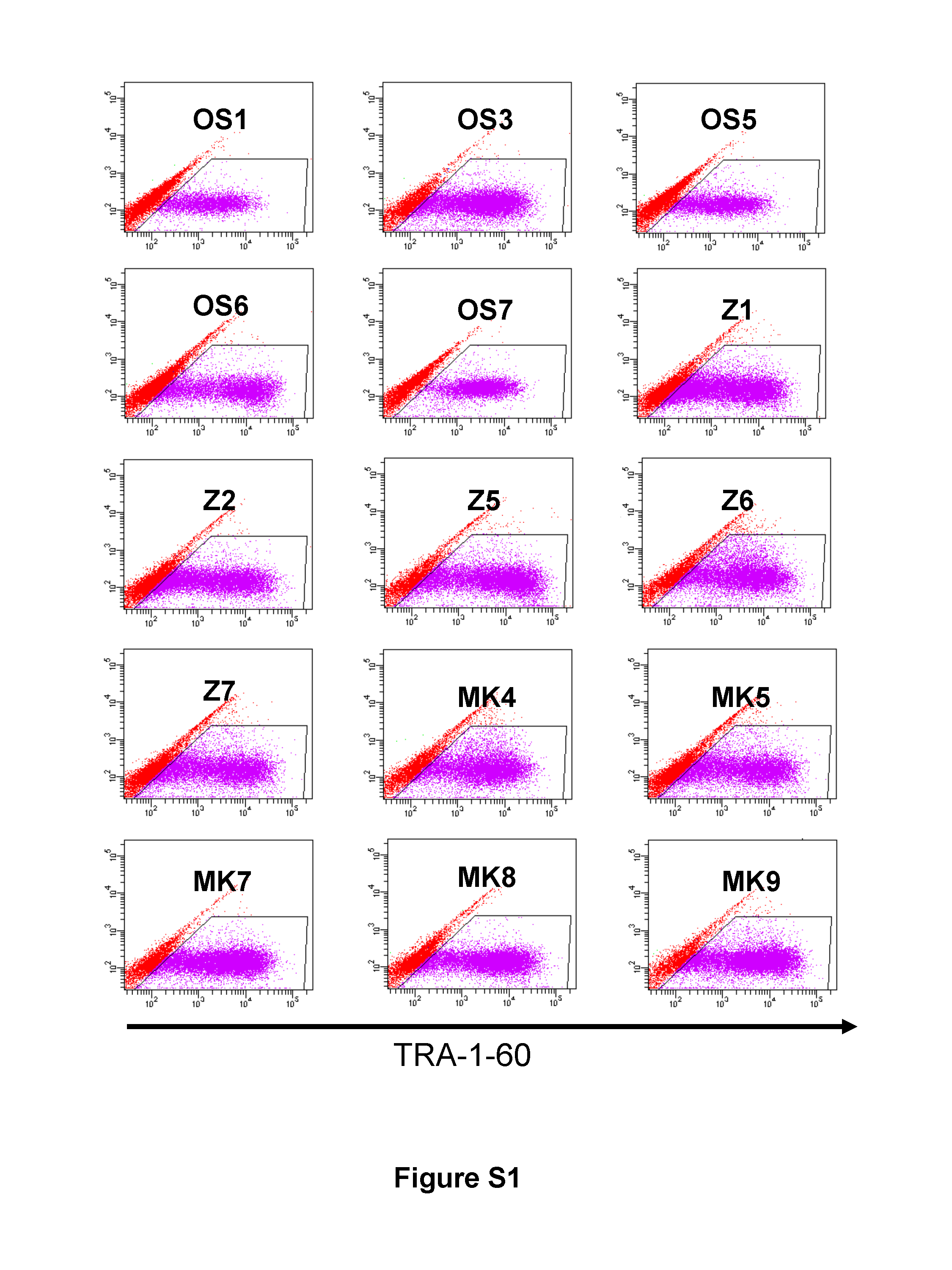

Supplement: Figure S1 — Flow cytometry analysis of CB iPSC lines. FACS diagrams show the expression of the pluripotency factor TRA-1-60 on bulk populations of 15 CB iPSC lines cultured with feeder support. OS, iPSCs generated with OS alone; Z, iPSCs generated with OSZ; MK, iPSCs generated with OSMK. (TIFF) [file pone.0059908.s001.tiff]

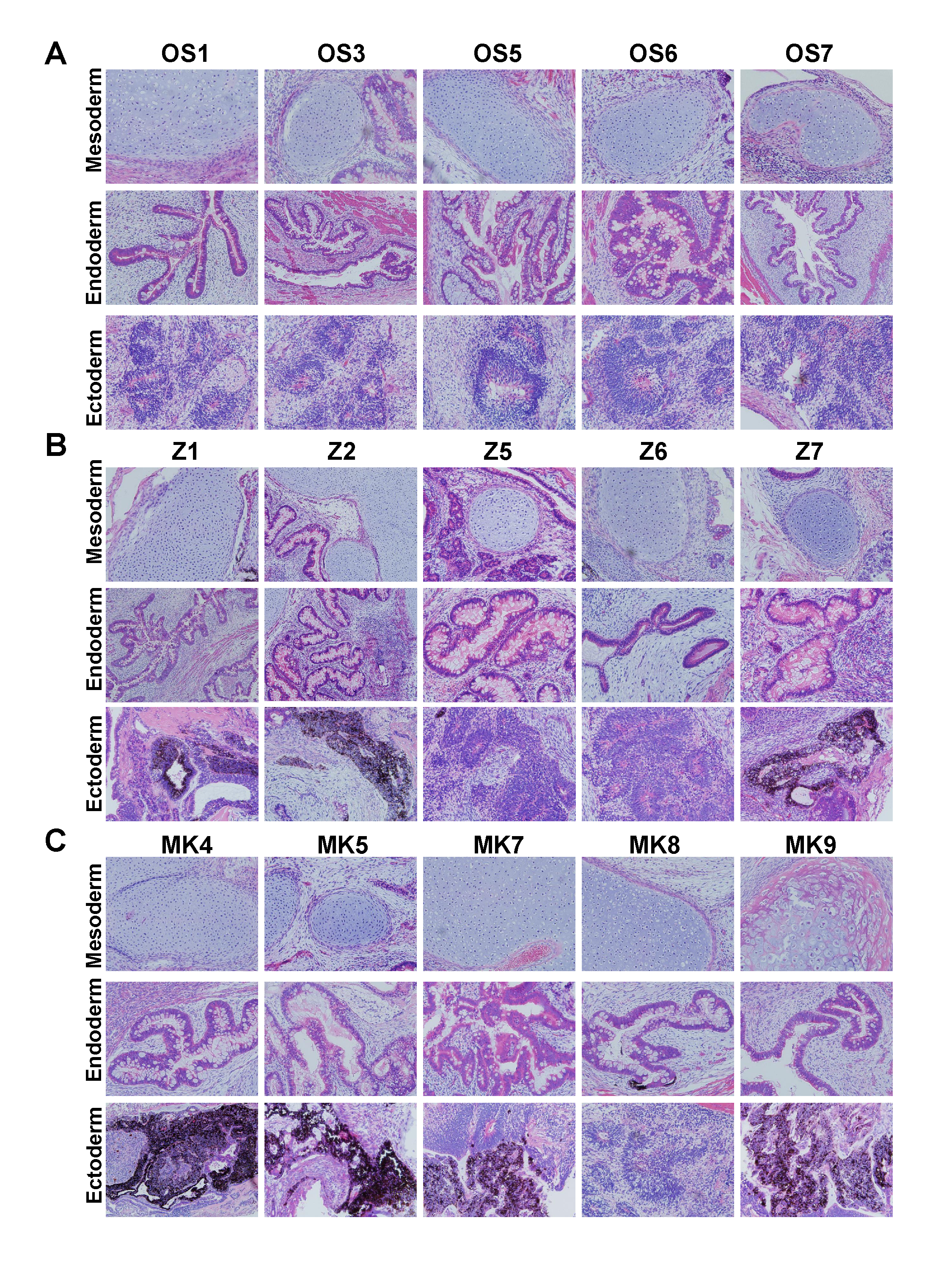

Supplement: Figure S2 — Teratoma formation from CB iPS cells. iPS cells were subcutaneously injected into NSG mice. After ∼2 months, the teratomas were analyzed by haematoxylin and eosin staining. (A) iPSCs generated with OS formed teratomas consisted of cartilage (mesoderm), gut-like structures (endoderm), and neurotubules (ectoderm). (B) iPSCs generated with OSZ (Z) formed teratomas consisted of cartilage (mesoderm), gut-like structures (endoderm), and neurotubules and pigmented epithelium (ectoderm). (C) iPSCs generated with OSMK (MK) lines formed teratomas consisted of cartilage (mesoderm), gut-like structures (endoderm), and neurotubules and pigmented epithelium (ectoderm). (TIFF) [file pone.0059908.s002.tiff]

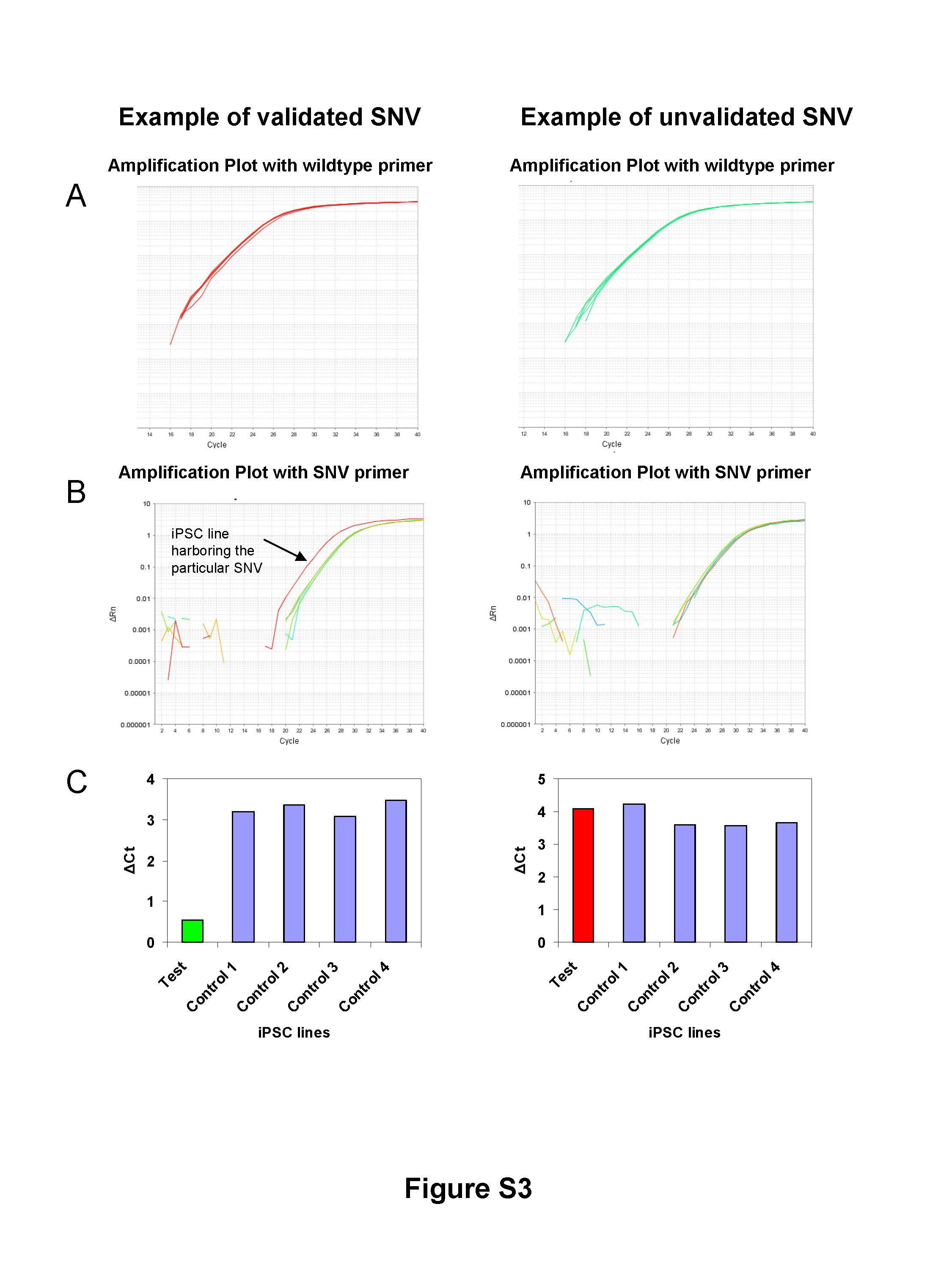

Supplement: Figure S3 — Validation of SNV by real-time PCR. (A). No obvious difference was observed when a pair of wildtype primers was use to amplify DNA from 5 iPSC lines. (B) When the primer harboring the SNV at the 3′ end was used, the sample DNA containing the particular SNV amplified more efficiently, leading to lower cycles. (C) In samples that ΔCt is substantially lower than the control, the SNV is validated (Left). However, in samples that ΔCt is not significantly lower than control (ΔΔCt <1), the SNV is not validated (Right). (TIFF) [file pone.0059908.s003.tiff]

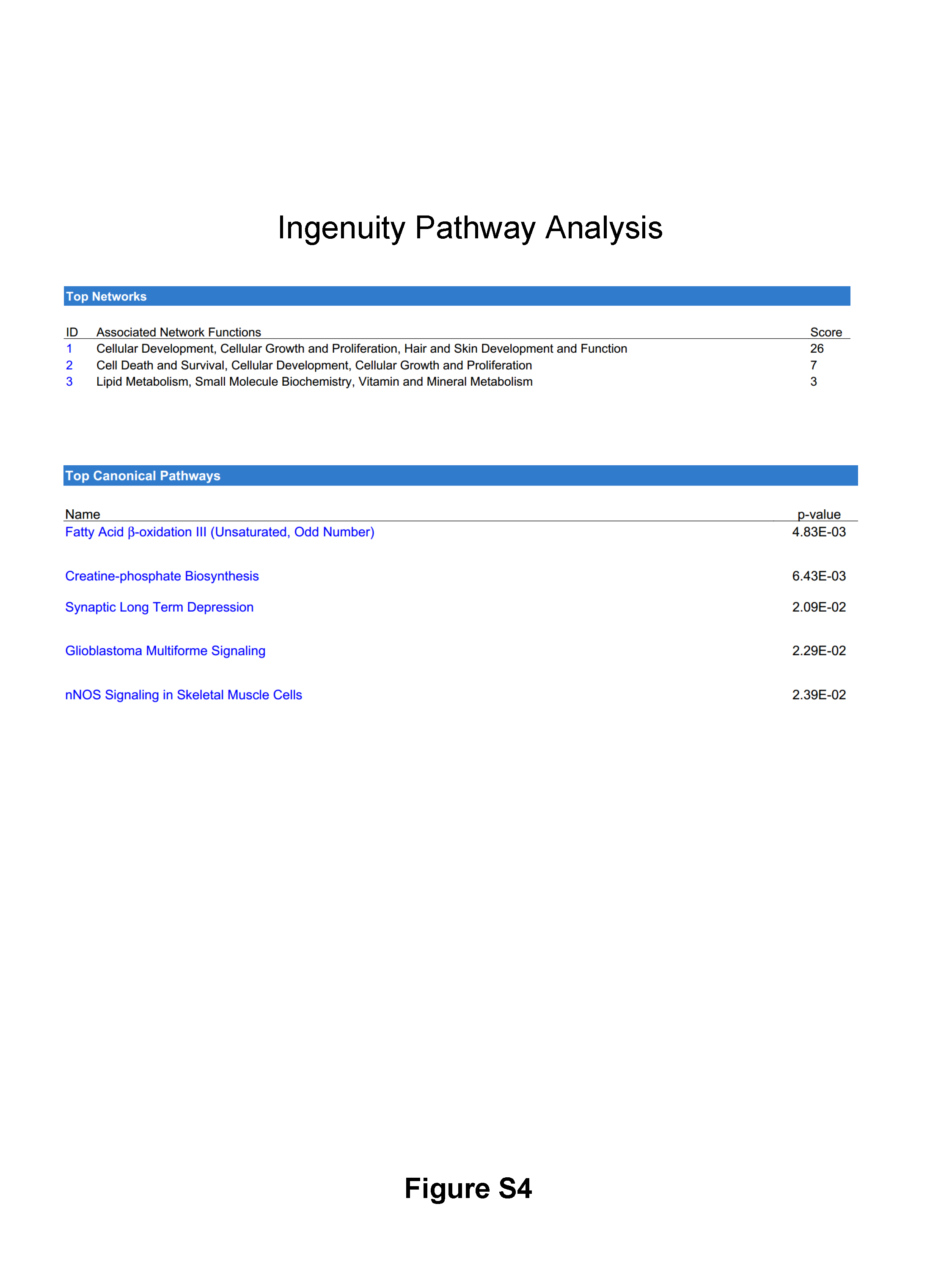

Supplement: Figure S4 — Ingenuity pathway analysis of all the 34 SNVs identified in 15 iPSC lines. (TIFF) [file pone.0059908.s004.tiff]
